# Fibrinogen concentrate versus placebo for treatment of postpartum haemorrhage: study protocol for a randomised controlled trial

**DOI:** 10.1186/s13063-015-0670-9

**Published:** 2015-04-17

**Authors:** Nadine Aawar, Raza Alikhan, Daniel Bruynseels, Rebecca Cannings-John, Rachel Collis, John Dick, Christopher Elton, Roshan Fernando, Judith Hall, Kerry Hood, Nicki Lack, Shuba Mallaiah, Helena Maybury, Jacqueline Nuttall, Shantini Paranjothy, Rachel Rayment, Alexandra Rees, Julia Sanders, Julia Townson, Andrew Weeks, Peter Collins

**Affiliations:** Innovation, Methodology and Engagement, South East Wales Trials Unit, Institute of Translation, Cardiff University School of Medicine, Neuadd Meirionnydd, Heath Park, Cardiff, CF14 4YS UK; Department of Haematology, Cardiff and Vale University Health Board, Cardiff, UK; Department of Anaesthetics and Pain Control, Cardiff and Vale University Health Board, Cardiff, UK; Department of Anaesthetics, University College Hospital, London, UK; Department of Anaesthetics, Leicester Royal Infirmary, Leicester, UK; Institute of Infection and Immunity, Critical Illness Research Group, Cardiff University School of Medicine, Cardiff, UK; Department of Obstetrics, University College Hospital, London, UK; Department of Anaesthetics, Liverpool’s Women’s Hospital, Liverpool, UK; Department of Obstetrics, Leicester Royal Infirmary, Leicester, UK; Institute of Primary Care and Public Health, Cardiff University School of Medicine, Cardiff, UK; Department of Obstetrics, Cardiff and Vale University Health Board, Cardiff, UK; Department of Women’s and Children’s Health, University of Liverpool, Liverpool, UK

**Keywords:** Postpartum haemorrhage, Blood transfusion, Fibrinogen concentrate, Fibtem

## Abstract

**Background:**

Postpartum haemorrhage (PPH) is a major cause of maternal morbidity. Bleeding is caused by a combination of physical causes, such as failure of the uterus to contract or operations, and is made worse by impairment of the blood clotting system. A number of studies have shown that low levels of the blood clotting factor fibrinogen are associated with progression of bleeding, the need for invasive interventions and transfusions of red blood cells and fresh frozen plasma (FFP). This trial will investigate whether early infusion of fibrinogen concentrate during a major PPH, with the aim of correcting a low fibrinogen to a level that is normal for delivery, based on the Fibtem test, reduces the total number of allogeneic blood products (red blood cells, FFP, cryoprecipitate and platelets) transfused after study medication until discharge, compared to placebo.

**Methods/design:**

This is a prospective, randomised, double-blind placebo controlled trial. Women will enter an observational phase and if their Fibtem levels fall they will be randomised in the interventional phase. A total of 60 women will be randomised and women are eligible for the trial if they meet all of the following inclusion criteria: age 18 years or over, gestation ≥24 + 0 weeks, haemorrhage of about 1500 ml and on-going bleeding without another complication or haemorrhage of about 1000 ml and caesarean section/uterine atony/placental abruption/placenta praevia/cardiovascular instability or microvascular oozing. Participants with a Fibtem A5 < 16 mm will be randomly allocated to receive either a bolus infusion of fibrinogen concentrate or placebo (isotonic saline). The dose of fibrinogen concentrate or placebo will be calculated based on the woman’s ideal body weight for height and the measured Fibtem A5 with the aim of increasing the Fibtem A5 to 23 mm.

**Discussion:**

The trial aims to provide evidence on the efficacy and safety of fibrinogen concentrate during acute bleeding in an obstetric setting.

**Trial registration:**

ISRCTN ref: ISRCTN46295339 (01.07.2013); EudraCT: 2012-005511-11 (28.11.2012), UKCRN ref: 13940.

## Background

Postpartum haemorrhage (PPH) is defined as blood loss of ≥500 ml after vaginal delivery or ≥1000 ml after caesarean section. Major PPH can be divided into moderate at 1000–2000 ml or severe at over 2000 ml. Severe PPH is also defined as a fall in haemoglobin of ≥40 g/l or the need for transfusion of 4 or more units of red blood cells (RBC) [1]. Massive PPH has been described as bleeds of more than 2500 ml and is associated with significant morbidity, even in resource-rich countries, including admission to high dependency and intensive care and emergency hysterectomy (6-8% of cases) [2].

PPH is a major cause of maternal morbidity and mortality. Worldwide PPH accounts for at least 30% of all maternal deaths [3], whilst in the UK massive PPH accounts for 75% of severe childbirth-related morbidity affecting 6:1000 deliveries each year [2]. The incidence of massive PPH has increased over the last 10 years in the UK and other countries [2,4-6] and outcomes have not improved over that time period. Deaths directly caused by obstetric bleeding in the UK have had an incidence between 0.33 and 0.93 per 100,000 deliveries over the last 15 years [7].

PPH results from a combination of physical causes such as trauma, uterine atony and placental complications and is exacerbated by haemostatic impairment. Haemostatic impairment is caused by a combination of consumptive and dilutional coagulopathies. This leads to low levels of fibrinogen early during PPH and before clinically significant depletion of other coagulation factors [8]. The low fibrinogen level results in the formation of poor quality clots and contributes to on-going bleeding. Bleeding is exacerbated by fibrinolysis (the breakdown of fibrin clots), which is often increased during PPH.

There are no high-quality data to guide the replacement of coagulation factors during PPH. Coagulation factors are often replaced with fresh frozen plasma (FFP) but there is limited evidence to support its efficacy. When as well as how much FFP to infuse in any clinical situation is debated [9]. Current guidelines recommend identical haemostatic support during PPH as for all other clinical situations of massive haemorrhage [1,10,11]. There is limited evidence to support these transfusion guidelines in any clinical situation and none in PPH; studies in this area have been highlighted as a priority [12].

Physiological changes in coagulation at the time of delivery mean that standard transfusion triggers, such as those used in trauma (and recommended in current guidelines), are unlikely to be applicable at the time of a major PPH. At term the prothrombin time (PT) and activated partial thromboplastin time (aPTT) are shorter and fibrinogen is higher than in the non-pregnant population. In addition, the PT and aPTT often remain normal despite very large bleeds. The current recommendation is to infuse FFP during PPH when the PT or aPTT is greater than 1.5 times normal [1], but this is not supported by any evidence and these coagulation abnormalities will be associated with established haemostatic failure and microvascular bleeding [11]. Audit data demonstrate that clinicians often do not follow these recommendations in clinical practice when dealing with severe PPH [8].

The fibrinogen level at the time of delivery is between 4–6 g/l compared to 1.5-4 g/l in the non-pregnant healthy population. Current recommendations are to maintain the fibrinogen level above 1 or 1.5 g/l [1,10,11]. During an on-going PPH it is not known whether fibrinogen should be replaced to a level appropriate to the non-pregnant population or a level appropriate for the time of delivery. There are data, however, suggesting that a fibrinogen level associated with the time of delivery might be more appropriate.

There are no systematic reviews of the use of fibrinogen concentrate in PPH; however, the fibrinogen level has been shown to be a useful predictor of progression from early to severe PPH in multiple studies. A fibrinogen level below 2.9 g/l, taken early during labour (before any bleeding has occurred), had an odds ratio of 19 for progression to PPH. In this study a fibrinogen of <2.9 g/l had a positive predictive value (PPV) of 39% for progression to PPH whilst a fibrinogen above 2.9 g/l had a negative predictive value (NPV) of 94% [13]. In other studies, a low fibrinogen level, taken early during a PPH, was predictive of the need for invasive procedures or infusion of RBC and FFP [14-16]. Charbit et al. found that a fibrinogen level below 2 g/l, taken during an on-going PPH, had a PPV of 100% for progression to severe PPH, defined in part as the need for at least 4 units of RBC, whilst a fibrinogen level above 4 g/l had a NPV of 85-90% [14]. Gayat et al. demonstrated that a fibrinogen level below 2 g/l, taken after a 1000 ml estimated blood loss, was an independent predictor for the need for an invasive procedure [15].

Retrospective data from 240 women experiencing a major PPH in a 6-month period, taken from the Cardiff Maternity Database, also found that fibrinogen was a useful predictor for progression to the need for RBC transfusion. The receiver-operator characteristic curve (ROC) area under the curve (AUC) (95% CI) for any RBC transfusion was 0.78 (0.74-0.83), the need for 4 units of RBC 0.8 (0.73-0.86) and for progression to 2500 ml blood loss 0.85 (0.78-0.93). In women with a fibrinogen level less than 3 g/l, 18/21 (86%) required RBC transfusion (median 3 units range 0–12) and 11 also required FFP. The mean (SD) allogeneic units (RBC + FFP + cryoprecipitate + platelets) infused was 5.8 (6.8). In contrast, in women with a fibrinogen level greater than 4 g/l (a normal level for delivery) 7/81 (8.6%) required RBC transfusion, a mean (SD) of 0.2 (0.6) units/woman, and none required FFP. These data have been confirmed in a prospective, consecutive cohort of 346 women in Cardiff experiencing a major PPH that showed that the ROC AUC (95% CI) for fibrinogen (*n* = 332) as a predictive biomarker for the need for 4 or more units of RBC transfusion was 0.77 (0.66-0.87).

Bleeding during PPH is often very fast and, in order to reduce the risk of progression from moderate to massive PPH, early intervention is required. If a low fibrinogen level is to be used as a trigger for intervention the result needs to be available to the clinician as soon as possible. Testing fibrinogen in the laboratory takes between 60 to 90 min and this is too long to be clinically useful in most cases of PPH. Furthermore, during a PPH the fibrinogen level may fall rapidly and so repeated measurements may be required to identify when a critically low level has been reached. A better strategy would be to use point-of-care testing to trigger a fibrinogen replacement and to guide the dose to be given.

It is not possible to replace fibrinogen to levels normally associated with delivery with FFP because the concentration of fibrinogen in FFP is less than 4 g/l. In addition, there is substantial delay between deciding to give FPP and/or cryoprecipitate and its infusion. This is because requesting FFP/cryoprecipitate from the laboratory, thawing it, issuing it and transporting it from the laboratory to the delivery suite usually takes around 60 min even with an efficient system. Fibrinogen concentrate (RiaSTAP® manufactured by CSL Behring, Marburg, Germany) can be used to rapidly replace fibrinogen to the pregnancy-related normal range [17] and will be used in this study. Anecdotal data suggest that fibrinogen concentrate may be useful as rescue therapy in managing severe hypofibrinogenaemia associated with massive PPH. In a number of case series, severe haemorrhage was clinically arrested or substantially improved when fibrinogen concentrate was used during severe and on-going bleeding [17-19].

A potential point-of-care surrogate test for the fibrinogen level is the Fibtem measured on the ROTEM® machine. The Fibtem assay gives results after 5 min (A5) and at the time of maximum clot firmness (MCF). Prospective data collected during major PPH in Cardiff have shown that the A5 and MCF are strongly correlated (r^2^ 0.93) but the A5 is available a median (IQR) 19 (13–24) min earlier than the MCF. The A5 is available on average 6 min after the start of the Fibtem assay. The A5 is between 1 and 2 mm lower than the MCF. The Fibtem A5 has been shown to correlate well with the laboratory-measured fibrinogen level during early PPH [20] and a result is available within 10–15 min after taking blood. The Fibtem assay has been used successfully to guide fibrinogen replacement with fibrinogen concentrate following complex cardiac surgery and was shown to have a large effect on bleeding and transfusion requirement [21].

In a Cardiff prospective observational study over a 12-month period of 346 analysable women experiencing a major PPH, 318 had a Fibtem performed and 332 had a Clauss fibrinogen (in press) (NB: the paper includes one more case than presented in this paper and hence the results are not identical). In this cohort the utility of Fibtem A5 and MCF to predict the need for red cell transfusion was investigated. For the need for any red cell transfusion, the ROC AUC (95% CI) for Fibtem A5 was 0.62 (0.55-0.69) and MCF was 0.62 (0.55-0.69), a result almost identical to that with the laboratory fibrinogen, 0.68 (0.61-0.75). Fibrinogen, Fibtem A5 and MCF had improved utility for predicting larger bleeds: for the need for 4 units of red blood cells the ROCs were 0.75 (0.64-0.87), 0.77 (0.67-0.87) and 0.78 (0.67-0.88) respectively. The results for the need for eight or more units of allogeneic products (red cells + FFP + platelets + cryoprecipitate) were 0.81 (0.70-0.92), 0.79 (0.67-0.91) and 0.79 (0.67-0.91) for fibrinogen, A5 and MCF respectively. This supports the potential use of Fibtem A5 or MCF instead of a laboratory fibrinogen level to guide treatment in this situation. Given that A5 and MCF give equivalent information and the A5 is available on average 19 min earlier than MCF, it allows earlier intervention during major PPH and hence is the preferred parameter to use for this study.

In the Cardiff prospective study there were 32 women with a Fibtem A5 below 16 mm (or a Clauss fibrinogen below 3 g/l) and on-going bleeding (>250 ml). Of these 27 (84%) required a red cell transfusion; mean (SD) number of units of red cells transfused was 4.1 (3.9). The mean (SD) number of units of allogeneic blood products was 8.2 (8.7). Of the 32 women, 7 also received between 3 and 7 g of fibrinogen concentrate as rescue haemostatic therapy.

Of the 288 women who had a Fibtem A5 of 16 mm or above or did not have on-going bleeding, and so would not have been randomised in the prospective study, 64 (22%) received a red cell transfusion, mean (SD) 0.55 (1.20 units/woman). The mean (SD) number of allogeneic units was 0.79 (2.19). The PPV (95% CI) for the need for a red cell transfusion of a Fibtem A5 below 16 mm in women with on-going bleeding was 84 (67–94)%. The NPV was 78 (73–82) %.

In the same study, of the 84 women with a Fibtem A5 of 23 mm or above, 16 (19%) received a red blood transfusion [median (range) 2 (2–5) units]. The mean (SD) number of units of red cells transfused was 0.44 (0.99) with a median (IQR) of 0 (0–0) units. The mean (SD) number of allogeneic units transfused was 0.58 (1.51) with a median (IQR) of 0 (0–0) units.

Although there is not a direct relationship between Fibtem and fibrinogen levels, in women experiencing major PPH, a Fibtem A5 of 16 mm is associated with a fibrinogen level below about 3 g/l and a Fibtem A5 of above about 23 mm with a fibrinogen above 4 g/l (prospective unpublished data from Cardiff and Huissoud 2009).

### Objective

The aim of this study is to investigate whether infusion of fibrinogen concentrate, based on the Fibtem A5 test, during a moderately severe PPH reduces the total number of allogeneic blood products transfused after study medication until discharged compared to placebo. The effect of fibrinogen on efficacy endpoints will be assessed as a secondary outcome.

## Methods and design

### Overview

**Figure 1:**
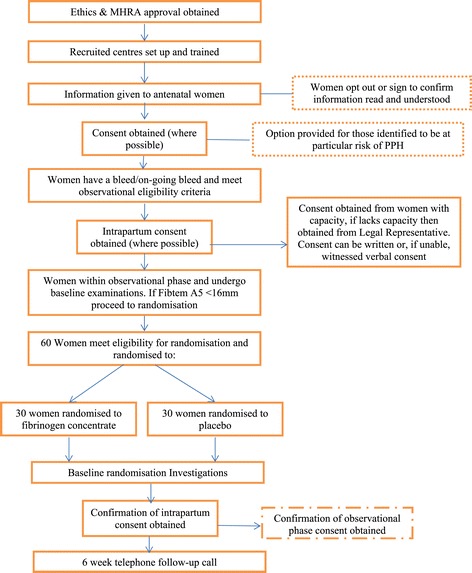
Trial schema.

This is a prospective, randomised, double-blind, placebo controlled trial. A total of 60 women will be included in the randomisation phase. The trial schema is shown in Figure 1.

### Inclusion criteria

- Age 18 years or over,
- Gestation ≥24 + 0 weeks
- Women should have any one of the following either before delivery or within 12 h after delivery: haemorrhage of about 1500 ml and on-going bleeding without another complication or haemorrhage of about 1000 ml and on-going bleeding with any of:

- o Caesarean section
- o Uterine atony
- o Placental abruption
- o Placenta praevia
- o Cardiovascular instability (arterial blood pressure below 90 mm/Hg and heart rate greater than 100 bpm)
- o Clinical observation of microvascular oozing

### Exclusion criteria

- Women who have documented that they do not want to participate in the study during the antenatal period
- Women declining infusion of red blood cells or blood components
- Known inherited bleeding disorder
- Placenta accreta diagnosed antenatally
- Women who have already received uterine brace sutures, uterine tamponade balloons, radiology intervention or hysterectomy before entering the study
- Clinical suspicion of amniotic fluid embolism
- Secondary postpartum haemorrhage (defined as abnormal bleeding starting between 24 h and 6 weeks after delivery).

### Sample size

On the basis of data prospectively collected in Cardiff in a study with the same entry criteria as this trial, the sample size was derived in the following way:

From a group of women with on-going bleeding (further bleeding of ≥250 ml after enrolment) and with a Fibtem A5 < 16 mm (representing women who would be the control/placebo group), the total number of allogeneic units (red cells + FFP + cryoprecipitate + platelets) transfused was positively skewed. The data were therefore transformed using the natural log (x + 1) transformation. This resulted in a more normal distribution with a mean [standard deviation (SD)] of 1.811 (1.004) on the log-transformed scale. Back-transforming this data gives a geometric mean of 5.12 units of allogeneic blood transfusions. Using the data from low-risk women with Fibtem A5 > 22 mm (representing what we would expect in the intervention group) then using the same transformation, the mean (SD) is 0.256 (0.539). Back-transforming gives a geometric mean of 0.30 units of allogeneic blood transfusions. This demonstrates a difference of 1.811-0.256 = 1.556 on the log-transformed scale.

A total of 60 patients can realistically be recruited. Therefore basing our sample size calculation on 27 per group (54 in total plus a 10% dropout), if the mean on the transformed scale in the intervention group is 1.036, this is the size of difference that we would be able to detect with power 80% at the two-sided 5% alpha level [22]. A transformed mean of 1.036 corresponds to a back-transformed ‘geometric mean’ of 1.81 allogeneic units. This is a 3.3 unit reduction, when measured as a simple difference on the original scale. The size of difference between control and intervention groups we might assume from this, when expressed on the log-transformed scale, is 1.811-1.036 = 0.775.

In the light of the difference found between the high- and low-risk groups (1.552), the assumption we are making for the new study still appears to be cautiously conservative. Thus we conclude that 27 individuals in each treatment arm (54 in total) would give 80% power to attain a difference of 3.3 total allogeneic units (0.775 on a log-transformed scale) at a conventional 5% alpha level. Assuming a 10% loss to follow-up, this will enable 60 women (30 per treatment group) to complete the trial and achieve the above numbers.

### Randomisation and blinding

Randomisation will be carried out using random permuted blocks. Computer-generated random numbers will be produced to select a block of allocations from the set of all possible permutations of allocations (on a 1:1 ratio) given a particular block size before the trial begins. Saint Mary’s Pharmaceutical Unit will undertake an exercise to validate the blinding process. A reproducible method that ensures all the fibrinogen does dissolve will be developed and detailed guidance produced that will form the basis of training the centre staff.

The fibrinogen concentrate will be supplied as vials of 1 g per bottle by CSL Behring to Saint Mary’s Pharmaceutical Unit (SMPU) in Cardiff. The placebo will be 50 ml of normal saline for each 1 g of fibrinogen. SMPU will supply each site with packs of blinded fibrinogen concentrate and placebo. At the time of randomisation the next consecutive numbered study pack will be opened. Black syringes will be used to draw the fibrinogen/placebo bottles with added diluents; the air will be expelled from the syringe into a bag of Gelofusine. SMPU has undertaken an exercise to validate the blinding process and ensure that all the fibrinogen dissolves.

### Observational phase

**Figure 2:**
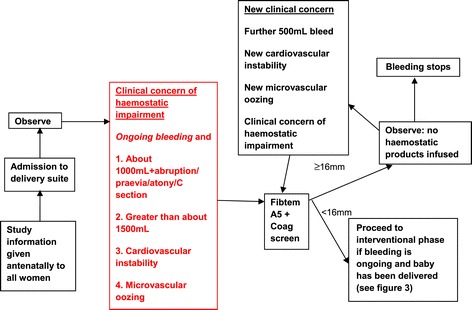
Observational phase.

When women fulfil the study entry criteria they will enter the observational phase (Figure 2). Standard obstetric procedures for managing PPH (except those relating to blood product usage) such as suturing, removal of retained products and surgical or tamponade treatments for resistant atony will be followed according to local and national guidelines and are at the discretion of the treating clinicians. All other management will follow standard local procedures and is at the discretion of the treating clinicians. When women fulfil the criteria:

- Blood will be taken for full blood count (FBC), coagulation screen and group and save/cross match and sent to the routine laboratory for analysis. These tests are part of standard care.
- At the same time as the routine blood tests, an extra 5-ml sample will be taken into a coagulation screen bottle and a Fibtem test will be performed on a ROTEM machine located in the delivery suite or obstetric theatre.
- A measurement will be made of the blood loss according to the procedures described below as soon as possible after study entry.

**Figure 3:**
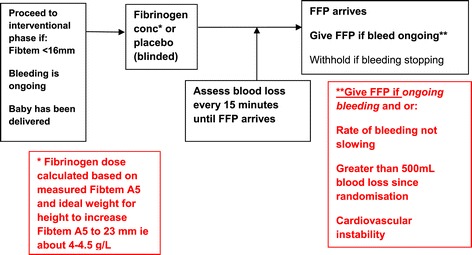
Interventional phase.

If the Fibtem A5 is less than 16 mm, the bleeding is on-going and the baby has been delivered, the woman will enter the interventional phase. If the Fibtem A5 is 16 mm or above further Fibtem assays may be performed after each 500 ml additional blood loss or for clinical concern. If any subsequent Fibtem falls below 16 mm the woman will enter the interventional phase (Figure 3).

### Interventional phase

The participants are assigned either to placebo (isotonic saline) or interventional drug (fibrinogen concentrate, Riastap® CSL Behring, Marburg, Germany). The dose of fibrinogen concentrate or placebo to be infused will be calculated based on the woman's ideal body weight for height and the measured FIBTEM A5 with the aim of increasing the Fibtem A5 to 23 mm. The dose will be calculated as: dose (g) = (23-actual Fibtem A5) × ideal body weight for height/140. The dose will be rounded to the nearest whole number of grams of fibrinogen and the maximum dose of fibrinogen concentrate or placebo will be 8 g. At the time of randomisation the following will be done:

- Four units of FFP will be ordered from the blood bank through routine procedures.
- A measurement of blood loss will be made at the time the FFP is requested.
- Tranexamic acid (1 g) will be given intravenously, if not already given. A further dose of 1 g can be given intravenously if bleeding continues after 1 h of the initial dose. Women should then receive tranexamic acid 8 hourly, either orally or intravenously, for a period of time determined according to local practice.
- At about 15 min after the infusion of fibrinogen/placebo a coagulation screen and sample for a Fibtem will be sent to the routine laboratory. These results cannot be accessed by study staff until after the decision has been taken to give or withhold the FFP because they are likely to unblind the study. A clinician can decide to access these results if the clinical situation requires it but if this happens the woman would not be included in the primary efficacy analysis.
- Blood loss will be measured every 15 min, if feasible, until the FFP arrives.
- An additional Fibtem and coagulation screen will be taken at 24 h after the study medication, if possible coinciding with a routine FBC.

The decision to give FFP will be taken by a team approach after consultation with either a consultant obstetrician and or a consultant obstetric anaesthetist. The FFP will not be given if the bleeding has stopped and/or the bleeding is slowing and the total blood loss is less than 500 ml.

The FFP will be given if the bleeding is on-going and there has been a further measured 500 ml blood loss since the infusion of fibrinogen/placebo, if the bleeding is on-going and the amount of bleeding has not reduced between the first and last 15-min observation period following randomisation, and if the bleeding is on-going and microvascular oozing or cardiovascular instability is clinically apparent.

#### Subsequent haemostatic therapy

If bleeding continues after the initial 4 units of FFP, the following protocol for blood product usage is suggested but clinicians may deviate from this protocol on a case-by-case basis for any reason:

Red blood cells will be transfused to maintain haemoglobin level above 8 g/dl. Near-patient testing of haemoglobin can be used.

The coagulation screen and FBC should be measured through the routine laboratory and repeated after transfusion of every 4 units of red blood cells or whenever clinically indicated. Point-of-care coagulation testing can be performed and the results acted on according to local practice.

FFP can be transfused in a 1:1 red cell:FFP ratio until bleeding has stopped, irrespective of coagulation results, and even if results are unknown.

In addition, if the PT or aPTT is greater than 1.5 times normal, a further 4 units of FFP can be given if bleeding is on-going. After the first 4 units of FFP, and if the fibrinogen level is less than 1.5 g/l, the cryoprecipitate (10 units) or fibrinogen concentrate at a dose of 60 mg/kg should be administered if bleeding is on-going. If the platelet count is less than 75 × 10^9^/l, 1 adult pool of platelets should be given.

#### Volume replacement

Crystalloid and colloid will be infused at the discretion of the treating clinicians. The colloid will be a gelatine-based product. The volume and time of infusions will be recorded retrospectively from the patient’s notes. The use of colloid hydroxyethyl starches will not be allowed because these interfere with the FIBTEM assay.

#### Postnatal thromboprophylaxis

All women will receive postnatal thromboprophylaxis with low molecular weight heparin. Low molecular weight heparin will be commenced as soon as is practical after bleeding has stopped.

### Setting, location and follow-up

The study will recruit from four centres in the UK. The criteria for selection are based on size of the centres (a minimum of approximately 5500 deliveries per year) and an interest in peripartum clinical studies. The study team at each centre will be based on delivery suite and consist of a lead obstetrician, obstetric anaesthetist and midwife. All obstetricians, obstetric anaesthetists and midwifes at each centre will be involved in the study.

Women recruited to the observational phase, but who do not enter the interventional phase of the study, will be followed up until hospital discharge. Women participating in the interventional phase will be followed up until 6 weeks following randomisation or death. At 6 weeks post-delivery, women who entered the interventional phase of the study will be contacted by telephone. The telephone interview will obtain data on maternal hospital readmissions, incidence of venous or arterial thrombotic events, and initiation and duration of breastfeeding.

### Outcomes and safety measures

The primary outcome is the total number of allogeneic blood products transfused after the study medication until discharge. The total number of allogeneic blood products transfused will be compared between the two arms. Secondary outcomes will be:

- the proportion of women receiving no allogeneic blood products until discharge and within 24 h after study medication,
- the number of units of red blood cells, FFP, platelets and cryoprecipitate transfused after study medication within 24 h and until discharge,
- the volume of cell salvaged blood transfused within 24 h after study medication and until discharge, the total number of units of red blood cells, FFP, platelets and cryoprecipitate transfused within 24 h after the study medication, the total number of units of red blood cells, FFP, platelets and cryoprecipitate plus 1 unit for every 250 ml cell salvage transfused within 24 h after study medication and until discharge, the measured abnormal blood loss within 24 h after study medication and until discharge, the proportion of women requiring cryoprecipitate or fibrinogen concentrate as subsequent therapy within 24 h after study medication and until discharge, the change in Clauss fibrinogen and Fibtem parameters before and 15 min and 24 h after the study medication, the proportion of women requiring invasive procedures (return to theatre, uterine brace sutures, uterine tamponade balloons, radiology intervention and hysterectomy) and the time of this intervention within 24 h after study medication and until discharge, incidence, duration and reasons for not exclusive breastfeeding.

The effects of fibrinogen concentrate infusion on the following safety endpoints will be explored: the proportion of women requiring high dependency and intensive care admission and length of stay, the total length of hospital stay and the incidence of clinically diagnosed arterial and venous thromboembolism within 6 weeks of study medication.

### Statistical methods and data analysis

#### Participant flow and recruitment

Summary statistics on eligibility, recruitment, withdrawal and dropout will be collated for both arms and will form the basis of the CONSORT flow diagram for clinical trial reporting [23], specifically, for each arm, participants randomly assigned, receiving intended intervention, completing the study protocol and analysed for the primary outcome. Any protocol deviations will be described with reasons as well as dates defining the period of recruitment and follow-up.

### Baseline data

Appropriate descriptive summaries and graphical illustrations of demographic and baseline clinical data (pre-study medication) will be presented. Descriptive summaries for selected variables will also be produced at site level. Analyses by study arm will be performed when the data collection for the final randomised patient has been completed and all follow-up data obtained. Baseline data will be used to check comparability between study arms and generalisability of the study population. There will be no formal testing of between-arm differences for any variables at baseline.

### Observational phase

Patients entering the observational phase but not randomised to receive fibrinogen/placebo because of a Fibtem A5 reading ≥16 will be described with regard to demographics and risk factors for PPH (such as pregnancy-related complications, labour and birth details, invasive procedures required to control PPH, bleed volume, transfusion requirement) and compared to the patients in the interventional phase. The whole cohort will be analysed to investigate risk factors for severe PPH and the need for blood transfusion.

### Interventional phase

The primary and secondary comparative analyses and presentation of this randomised trial will be in accordance with CONSORT guidelines and based on the intention-to-treat (ITT) principle without imputation of missing values (complete case population). The intention-to-treat population uses all randomised participants in the groups they were randomised to regardless of the intervention received and the complete case population restricts the population to include all randomised participants with complete data and follow-up. ITT analysis with missing values imputed will be undertaken as a sensitivity analysis.

### Primary analysis

For those who enter the interventional phase of the study, the primary outcome will compare the total number of units of allogeneic products (red blood cells, FFP, cryoprecipitate, and platelets) transfused after study medication until discharge from hospital between women who received fibrinogen concentrate and placebo. A Poisson regression model will be used with due emphasis placed on confidence intervals for the between-arm comparisons with an investigation of site-level clustering. The primary analyses will also be repeated controlling for any baseline imbalances. While the main analyses will be based on ITT without imputation, we will undertake sensitivity analyses in order to estimate the effect of those randomised to fibrinogen but not receiving it using complier average causal effect (CACE) estimates, implemented in Stata using instrumental variable regression [24].

### Secondary analysis

Secondary analysis includes investigating the effect of fibrinogen on:

- the total number of units of allogeneic products (red blood cells, FFP, cryoprecipitate and platelets) transfused within 24 h after study medication;
- the individual number of units of red blood cells, FFP, platelets and cryoprecipitate transfused after study medication within 24 h and until discharge;
- the total number of units of allogeneic products red blood cells, FFP, cryoprecipitate and platelets) plus 1 unit for every 250 ml cell salvage transfused within 24 h after study medication and until discharge,

and will be analysed using similar methods to those used in the primary analysis.

The effect of the intervention on the proportion of women receiving no allogeneic blood products, requiring cryoprecipitate or fibrinogen concentrate as subsequent therapy, and requiring invasive procedures, all within 24 h after the study medication and until discharge, will be examined by fitting logistic regression models again investigating for possible site-level clustering. The effect of fibrinogen on the incidence of exclusive breastfeeding will be explored using the same method.

Secondary outcomes also examined will be (1) the volume of cell-salvaged blood transfused within 24 h after the study medication and until discharge (2) and the measured abnormal blood loss within 24 h after study medication until discharge. These outcomes will be examined using linear regression models. Each of the regression model assumptions will be checked including but not limited to examining the distribution of the residuals and a plot of the residuals vs. predicted values. Similarly, Clauss fibrinogen and Fibtem parameters at 15 min and 24 h after the study medication will be examined between arms using a linear regression model adjusting for baseline measurements. The duration to (1) stopping breastfeeding or expressing breast milk and (2) any invasive procedure within 24 h after study medication and until discharge between arms will be examined using Cox’s proportional hazards model. Reasons for not exclusive breastfeeding will also be explored by using ordinal regression model.

The effects of fibrinogen concentrate infusion on the following safety endpoints will be examined: the proportion of women requiring high-dependency and intensive care admission and length of stay, the total length of hospital stay and the incidence of clinically diagnosed arterial and venous thromboembolism within 6 weeks of the study medication.

### Consent

All women at each study centre will receive an information leaflet in the antenatal period describing the study and consent process. Women will have the opportunity to indicate they would not wish to participate in the study at this stage. Consent will be obtained prior to birth for women planning an elective caesarean section or considered by a clinician to be at high risk of PPH, and immediately prior to study entry for other women. All women who give consent during a PPH, or consent is given on their behalf by a representative, will be seen by a member of the study team within 24 h of enrolment, or as soon as the woman’s condition permits, to discuss their participation in the study and obtain consent for use of the collected data.

### Research governance/ethical considerations

Full ethical approval for this study has been obtained from the Research Ethics Committee for Scotland (Ref: 13/SS/0008). The study will be conducted in compliance with the following:

- Declaration of Helsinki (Seoul, 2008)
- ICH Harmonised Tripartite Guideline for Good Clinical Practice.
- The Medicines for Human Use (Clinical Trials) Regulations 2004 (Statutory Instrument 2004 No. 1031) as amended by the Medicines for Human Use (Clinical Trials) Amended Regulations 2006 (Statutory Instrument 2006 Nos. 1928 and 2984) and Amended Regulations 2008 (Statutory Instrument 2008 No. 941).
- Research Governance Framework for Health and Social Care (Welsh Assembly Government 2nd Edition, September 2009, and Department of Health 2nd Edition, July 2005).

Two external bodies, a Data Monitoring and a Trial Steering committee will monitor the study progress.

## Discussion

Postpartum haemorrhage is a major cause of maternal morbidity and mortality. Fibrinogen appears to be a useful biomarker to predict the progression from moderate to massive PPH and the need for blood transfusion; however, it is not known whether replacement of fibrinogen to levels normal for the peripartum period (4–6 g/l), during an on-going PPH, would reduce bleeding and the need for transfusion. Although fibrinogen concentrate is not licensed for use in obstetric haemorrhage in the UK, it has been used off-label to correct hypofibrinogenaemia [17] and is the recommended treatment in other countries [25].

This trial is designed to detect haemostatic impairment rapidly and early during major PPH, identified by a near patient Fibtem A5 test below 16 mm, and replace fibrinogen to a level within the lower end of the normal range associated with delivery. Laboratory tests of fibrinogen take about 60 min to become available, and this is usually too slow to inform clinical intervention. The Fibtem A5 result is available within about 10–15 min of venesection and so can be used for decisions on therapeutic intervention. The decision to use the criteria of on-going bleeding and a low for delivery Fibtem A5, rather than a more pragmatic approach of randomising all women with a specific estimate blood loss, was made to minimise the chance of women being exposed to unnecessary IMP because many women in that situation would have a fibrinogen level above 4 g/l and therefore be unlikely to respond to a higher level The amount of fibrinogen concentrate or placebo given to all participants allocated to the intervention group will be based on the woman’s ideal weight for height and the Fibtem level. The dose will be rounded to the nearest whole number of grams of fibrinogen and the maximum dose of fibrinogen concentrate or placebo will be 8 g. The dose of fibrinogen is designed to increase the Fibtem A5 and Clauss fibrinogen to the lower end of the normal range for delivery. This level has been shown to be associated with a reduced requirement for blood products. This approach was preferred to the pragmatic approach of giving a standard dose of fibrinogen because this would be likely to be associated with both under- and over-dosing. The lower end of the normal range has been targeted to minimise the risk of thrombosis.

The study is designed to be blinded until the decision to give the first 4 units of FFP has been taken and hence the Fibtem A5 taken 15 min after IMP is not known to the clinicians managing the case. The primary endpoint is a comparison of allogeneic products between the two arms and knowledge of whether fibrinogen or placebo had been given is likely to affect the decision to give FFP, cryoprecipitate and fibrinogen. After the first FFP has been given and if the bleeding is on-going, clinicians need to know the fibrinogen level to optimally manage the case. At this point Fibtem A5 and Clauss fibrinogen tests can be performed that are available to clinicians. This will have the effect of partially unblinding the study because clinicians will know whether the Fibtem has increased or not. This partial unblinding was deliberately included in the study for women with on-going bleeding for safety reasons.

The primary objective is to investigate whether infusion of fibrinogen, based on a Fibtem A5 test, during a moderately severe PPH reduces the total number of allogeneic blood products (FFP, red blood cells, platelet concentrates and cryoprecipitate) transfused after study medication until discharge compared to placebo. This was chosen because the alternative primary endpoint of estimated blood is very difficult to measure accurately and reproducibly, especially in a multicentre study.

At the time of a PPH women may not have full capacity to give informed consent because of factors such as pain, altered levels of consciousness due to drugs and blood loss and anxiety. In addition, some clinical situations are so acute that there will be insufficient time to read and understand a full patient information sheet. The consent procedures adopted in this trial are based on recommendations of the Royal College of Obstetricians and Gynaecologists for obtaining consent to research in labour, which recommend against taking antenatal consent from all women if the likelihood of the event is less than one in ten deliveries [26].

## Trial status

The trial was initiated in June 2013. By November 2013, all four centres were screening and recruiting participants. By March 2014, 16 participants had been included in the randomisation phase.

## Abbreviations

APTT: Activated partial thromboplastin
AVC: Area under the curve
FBC: Full blood count
FFP: Fresh frozen plasma
ICH: International Conference of Harmonisation
IQR: Interquartile range
ITT: Intention to treat
MCF: Maximum clot firmness
NPV: Negative predictive value
PPH: Postpartum haemorrhage
PPV: Positive predictive value
RBC: Red blood cells
RCOG: Royal College of Obstetricians and Gynaecologists
ROC: Receiver operator characteristic curve
